# Protein transduction domain of translationally controlled tumor protein: characterization and application in drug delivery

**DOI:** 10.1080/10717544.2022.2122636

**Published:** 2022-09-14

**Authors:** Jeehye Maeng, Kyunglim Lee

**Affiliations:** Graduate School of Pharmaceutical Sciences, College of Pharmacy, Ewha Womans University, Seoul, Republic of Korea

**Keywords:** Cell-penetrating peptide, drug delivery, protein transduction domain, translationally controlled tumor protein, translocation mechanism

## Abstract

Our research group reported in 2011 the discovery of a novel cell-penetrating moiety in the N-terminus of the human translationally controlled tumor protein (TCTP). This moiety was responsible for the previously noted membrane translocating ability of purified full-length TCTP. The hydrophobic nature of TCTP-derived protein transduction domain (TCTP-PTD) endowed it with unique characteristics compared to other well-known cationic PTDs, such as TAT-PTD. TCTP-PTD internalizes partly through lipid-raft/caveolae-dependent endocytosis and partly by macropinocytosis. After cell entry, caveosome-laden TCTP-PTD appears to move to the cytoplasm and cytoskeleton except for the nucleus possibly through the movement to endoplasmic reticulum (ER). TCTP-PTD efficiently facilitates delivery of various types of cargos, such as peptides, proteins, and nucleic acids in vitro and in vivo. It is noteworthy that TCTP-PTD and its variants promote intranasal delivery of antidiabetics including, insulin and exendin-4 and of antigens for immunization in vivo, suggesting its potential for drug delivery. In this review, we attempted to describe recent advances in the understanding regarding the identification of TCTP-PTD, the characteristics of its cellular uptake, and the usefulness as a vehicle for delivery into cells of a variety of drugs and macromolecules. Our investigative efforts are continuing further to delineate the details of the functions and the regulatory mechanisms of TCTP-PTD-mediated cellular penetration and posttranslational modification of TCTP in physiologic and pathological processes. This is a review of what we currently know regarding TCTP-PTD and its use as a vehicle for the transduction of drugs and other molecules.

## 1. Introduction

Biological membranes consist of lipid bilayers in which proteins are embedded. These membranes separate the cytoplasm from the extracellular milieu by regulating the movement of molecules across the membrane. These selectively permeable barriers limit the internalization of external molecules such as specific medications and drugs, unless specific mechanisms such as endocytosis are involved (Joliot & Prochiantz, 2004). As the target molecules for specific medications need to enter the interior of the cells to be effective, there has been great research interest in vehicles that enable the efficient intracellular delivery of therapeutic macromolecules. The discovery of peptide moieties that can translocate into the cells opens up new ways to deliver various types of potential medicinal cargoes, such as small and large molecules including chemicals, peptides, proteins, antisense nucleotides, and liposomes. These peptide moieties are generally called protein transduction domains (PTDs) or cell-penetrating peptides (CPPs).

PTDs are a class of the short peptides (generally <20 amino acids), which can translocate across the biological membranes of live cells and carry heterologous cargos in vitro and in vivo. In the late 1980s, it was first shown that the transactivator of transcription (TAT) protein of HIV-1 was internalized into cells and translocated into the nucleus to activate human immunodeficiency virus type I (HIV-1) transcription (Frankel & Pabo, 1988). Since the discovery of the TAT-PTD, a wide variety of peptide sequences of natural origins or newly synthesized, such as polyarginine, transportan, penetratin, MAP, and Pep-7, that were able to translocate small and big molecules into cells have been reported (Joliot & Prochiantz, 2004; Skotland et al., 2015). The majority of PTDs are cationic molecules, enriched with positively charged amino acid residues such as lysine and arginine (Futaki et al., 2001) and amphipathic molecules with α-helix structure (Drin et al., 2001).

As mentioned, the first and most studied PTD was TAT-PTD, derived from a transcription factor of HIV-1 that activates the transcription of HIV-1 in infected host cells. The report on TAT peptide-mediated delivery of proteins established the therapeutic significance of PTD’s application such as TAT peptides (TAT_1-72_, TAT_37-72_), which when linked to large proteins facilitated the introduction of the linked complexes into the cells (Fawell et al., 1994). This was followed by the identification of a small peptide component of TAT (TAT-PTD) (Vivès et al., 1997) that has the ability to transport large proteins in vivo (Schwarze et al., 1999). Mutational analysis of TAT-PTD (TAT_48-57_, GRKKRRQRRR) mapped to a peptide with 9 amino acid residues (TAT_49-57_, RKKRRQRRR) sufficient for internalization and delivery of exogenous protein into cells (Park et al., 2002).

As with other PTDs, the discovery of TCTP-PTD originated from our unexpected observation that full-length translationally controlled tumor protein (TCTP) protein can translocate into cells. The protein was called ‘TCTP’ (Gross et al., 1989) because the cDNA sequence coding for it was from a human tumor and its expression was regulated at the translational level (Bommer & Thiele, 2004). It was soon found that TCTP is ubiquitously expressed in all eukaryotes with a high degree of conservation through phylogeny and that its expression is regulated not only at the translational but also at the transcriptional level by a great variety of stimuli (Bommer & Thiele, 2004) and that TCTP plays a multifaceted role through interactions with a multitude of partners such as chaperone proteins, nucleic acid-binding proteins, and cytoskeletal proteins (Li & Ge, 2017). TCTP is a Ca^2+^- and microtubule-binding protein and is involved in intracellular processes including growth and development, cellular stress responses, protein degradation, and autophagy as well as in extracellular cytokine-like functions (Bommer & Thiele, 2004; Bommer & Kawakami, 2021). TCTP is alternatively called ‘histamine-releasing factor (HRF)’ because of its role in inducing histamine release from the cells, which enables it to influence in human allergic responses (MacDonald et al., 1995; Kim et al., 2009).

In this review, we describe the efforts of our laboratory in the identification, characterization of TCTP-PTD and its utility as a vehicle for the transport of molecules into cells. TCTP-PTD is a novel type of PTD with a hydrophobic sequence originating from human protein, which exhibits behavior quite different from that of the better known TAT-PTD. Our exhaustive in vivo studies using TCTP-PTD-cargo complexes linked with various types of cargoes indicate that TCTP-PTD has the potential for use in drug delivery. This review also discusses the biological implications and roles of TCTP-PTD and offers some speculation on its potential usefulness.

## 2. Discovery of TCTP-PTD as the cell penetrating domain of TCTP

### 2.1. Discovery of TCTP-PTD as the cell penetrating and molecule-transducing domain of TCTP

**Figure 1.**
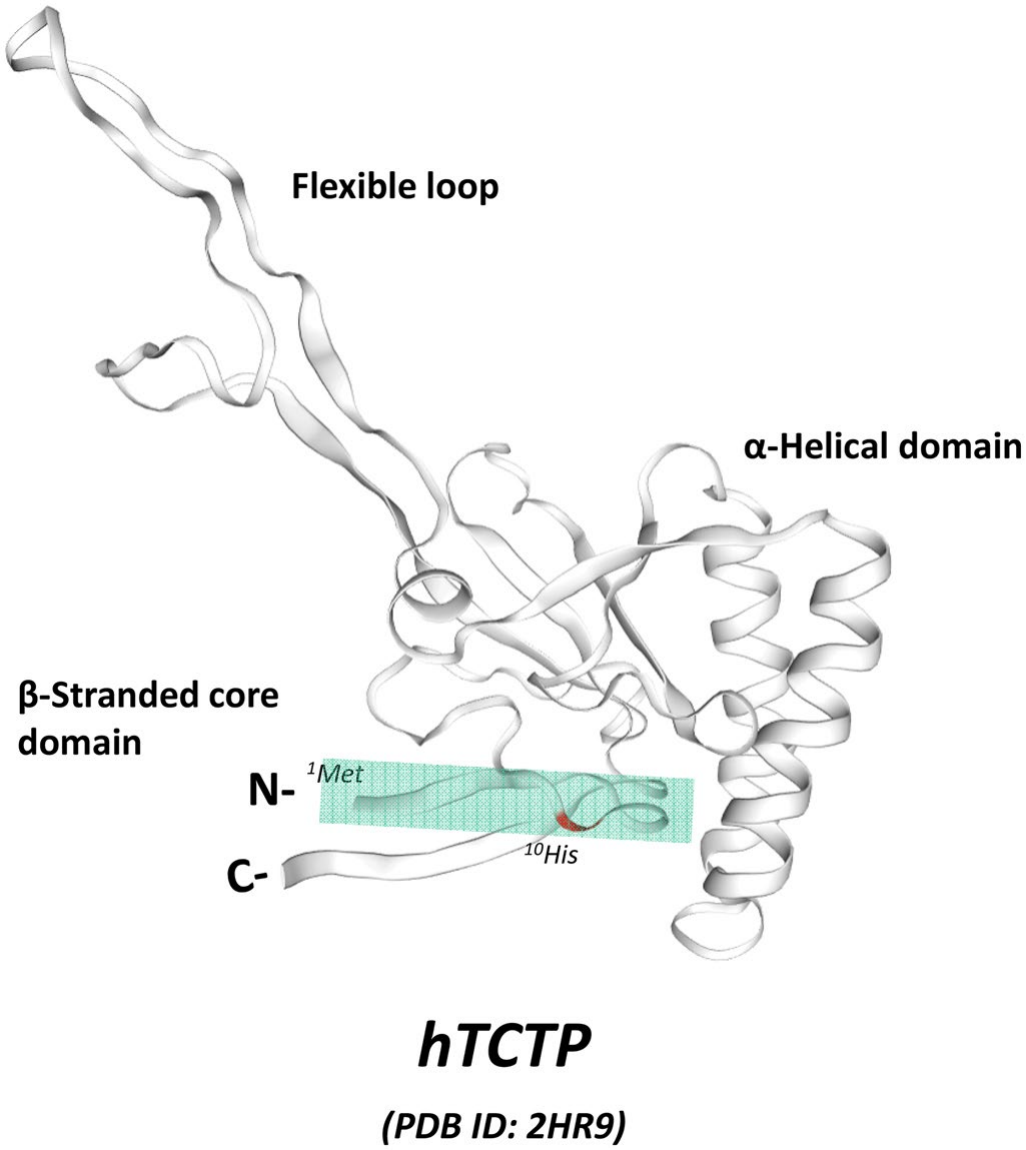
The N-terminal location of TCTP-PTD in the solution structure of human TCTP (hTCTP). NMR structure of hTCTP (Feng et al., 2007) was obtained from the ExPasy website (PDB ID: 2HR9, https://swissmodel.expasy.org), SWISS-MODEL repository (Bienert et al., 2017). The major domains of hTCTP is indicated such as flexible loop, α-helical domain, and β-stranded core domain. TCTP-PTD (1-MIIYRDLISH-10) is located at N-terminus of TCTP, as highlighted with colored box.

The discovery of TCTP-PTD occurred with the fortuitous observation of the internalization of purified full-length TCTP protein into RBL-2H3 cells in a confocal microscopic experiment (Kim et al., 2011b). We confirmed that what we observed was true internalization of TCTP into the cells and not a fixation artifact. Through a mutational analysis to locate the potential PTD region in full-length TCTP (1-172) using several deletion mutants, including TCTP-1-38, TCTP-39-110, TCTP-111-172, TCTP-11-172, and TCTP-35-172, we concluded that a 10 amino acid moiety at the N-terminus (1-MIIYRDLISH-10) is responsible for cellular uptake of full-length of TCTP (Figure 1). The cargo delivery capability of TCTP-PTD into various types of cells and into the tissues of mice was confirmed using both TAMRA-labeled and β-galactosidase (β -Gal)-fused forms of PTD (Kim et al., 2011b). The key characteristic of the TCTP-PTD is that it is quite different from other well-known cationic or amphipathic PTDs in terms of its unique sequence and behavior, as described in the following sections.

### 2.2. The characteristics of translocation of cargoes by TCTP-PTD

Our studies collectively showed significant differences in transduction kinetics between TCTP-PTD and TAT-PTD. TCTP-PTD efficiently internalizes into cells at relatively higher doses in a delayed time periods, while the translocation of TAT-PTD occurs at relatively lower doses during the first 1-2 h (Kim et al., 2011b). This phenomenon was reproduced for PTD-cargo delivery in parallel experiments using PTD-β-Gal fusion proteins (Kim et al., 2011b). The delivery of cargos by TCTP-PTD requires a longer treatment time and higher doses compared to TAT-PTD, which suggests that the two vehicles use different mechanisms. Also TCTP-PTD appears to be a better transducer in vivo than TAT-PTD but not in vitro. For example, our studies using cargos such as Cu,Zn-superoxide dismutase (SOD) and KLA showed similar patterns, indicating better in vivo delivery efficiency of TCTP-PTD compared to TAT-PTD (Kim et al., 2011a; Lee et al., 2011). It can be speculated that highly cationic peptides may efficiently translocate into single cell layers via interactions with charged heparan sulfate than hydrophobic PTDs whereas the hydrophobicity of TCTP-PTD may enable it to easily penetrate through tissues comprising multilayered cells in vivo.

We found that injection of TCTP-PTD fused with β-Gal into mice resulted in delivery into the major organs, including the liver, spleen, kidney, lungs and heart, but rarely to the brain (Kim et al., 2011b). However, TAT-PTD-β-Gal fusion protein is reported to be delivered to all tissues including the brain, suggesting a penetration through the blood-brain barrier (BBB) (Schwarze et al., 1999). Our later study with TCTP-PTD fused to SOD revealed the cargo-dependent effect on brain permeability of TCTP-PTD. TCTP-PTD-SOD was efficiently delivered into the hippocampal region, indicating the BBB-translocating ability (Lee et al., 2011).

### 2.3. Structural requirements of TCTP-PTD that promote cellular penetration and translocation of molecules into cells

Mounting evidence shows that PTDs generally have certain common features in sequences and secondary structures, such as polycationic residues and hydrophobic core domains, and α-helical or amphipathic structures to be efficient vehicles for delivery. For example, the highly cationic TAT-PTD (RKKRRQRRR) has six arginine and two lysine residues, all of which are hydrophilic basic residues. The mutation of any one of those residues largely impairs the energy-dependent cellular uptake of PTDs (Wender et al., 2000). As for penetration, the permeability is strongly dependent on the central hydrophobic core including ^6 ^W and ^7 ^F (Derossi et al., 1994; Wender et al., 2000). Generally, hydrophobic residues, including phenylalanine and tryptophan and positively charged amino acid, such as arginine and lysine, are the frequently used amino acids in the artificial design of PTD sequences (Lönn & Dowdy, 2015).

Interestingly, a unique sequence of TCTP-PTD is that it is mainly composed of hydrophobic cores of ^1^MII and ^7^LI, and partly of 5 charged or polar amino acids, including ^4^YRD, ^9^S, and ^10^H. In studies designed to establish the role of, or need for, each residue and the structural requirements of TCTP-PTD for cellular uptake, TCTP-PTD derivatives with deleted or substituted sequences were tested for their translocating activity (Kim et al., 2011c). These studies revealed that the hydrophobic nature of TCTP-PTD, especially at WT-I (the mutated position at first residue of wild type sequence of TCTP-PTD) through IV, and WT-VI through VIII is important for cellular penetration (Kim et al., 2011c). For example, the substitute ^6 ^D with ^6 ^A in TCTP-PTD showed enhanced cellular uptake (Kim et al., 2011c). They also revealed that hydrophobic residues may play a role in supporting the interaction of lipid-raft in the biological membrane at the initiation phase of cell entry.

Further studies after the truncation of amino- or carboxy-terminal provided information for minimum length of sequence for cell penetration. It was previously reported that the minimum length for TAT-PTD (48-57) for transduction was amino acids 49-57 (Wender et al., 2000; Park et al., 2002), indicating that ^48^Gly residue at N-terminal of TAT-PTD is not essential for efficient cellular entry. In our study, the deletion variant devoid of the first residue of TCTP-PTD (TCTP 2-10) showed a considerable reduction in cellular translocation whereas the C-terminal deletion of one residue (TCTP 1-9) retained comparable activity to that of TCTP-PTD. This demonstrates that first residue (^1^Met) at N-terminus (Kim et al., 2011c) and at least nine residues of N-terminus of TCTP (1-MIIYRDLIS-9) are critical requirements for cellular permeability (Kim et al., 2011c).

## 3. Modification of TCTP-PTD to optimize its ability to penetrate cells

Thirty-five variants of TCTP-PTD were prepared by chemical modification of its amino acid sequences with the goal of improving its ability to penetrate cells and analyzed for their characteristics including solubility to penetrate HeLa cells (Kim et al., 2011c). Addition of Lys residue at the C-terminus of TCTP-PTD facilitated its translocating activity (Kim et al., 2011c). Some variants to which one or two Lys residues were added at the C-terminus of the peptide showed improved solubility. Replacement of Met with Lys at WT-I enhanced the translocation, consistent with the mutational analysis showing that the characteristics of first residue is critical for translocation (Kim et al., 2011c). Arg residue at WT-V was found necessary for the solubility of hydrophobic TCTP-PTD. The presence of Arg rather than Lys or Ala at WT-V was found a beneficial for efficient translocation (Kim et al., 2011c). The guanidine head group of Arg in PTD was reported to take part in the hydrogen bonding between the lipid bilayer and Arg of peptide (Goun et al., 2006).

These TCTP-PTD modification studies were followed by cell penetration and cell cytotoxic assays, among others, and several modified derivatives were selected for their enhanced cell penetration efficiency, and concentration-dependent toxicity and compared with those of TAT-PTD and TCTP-PTD in HeLa cells. In our later studies, additional optimized and modified variants from TCTP-PTD13 (MIIFRALISHKK), such as TCTP-PTD13M1 (MIIFRLLISHKK), TCTP-PTD13M2 (MIIFRLLASHKK), and TCTP-PTD13M3 (MIIFRLLAYHKK) were specifically developed for their ability to perform intranasal drug delivery (Bae et al., 2018a).

## 4. The mechanism of TCTP-PTD translocation

The cellular translocation processes facilitated by PTDs largely comprise of sequential steps such as (i) interaction and association of PTD with the membrane, (ii) internalization by endocytosis, and (iii) intracellular trafficking, including endosomal escape into the cytoplasmic compartment (van den Berg & Dowdy, 2011). To perform their roles as cargo, they need to escape from intracellular vesicles and be released into the cytoplasm (van den Berg & Dowdy, 2011).

It is widely accepted that the internalization mechanism of PTDs and the progress of the uptake process are influenced by multiple elements, such as the nature of PTD and attached cargoes, linking methods, and experimental details such as the cell type, treatment conditions such as concentrations of reagents used and protocols applied during the investigation (van den Berg & Dowdy, 2011). There is no consensus on the exact molecular details for the precise internalization processes (Zhang et al., 2012) except that the majority of PTDs transduce cells by endocytosis or direct penetration (Ruseska & Zimmer, 2020). What is currently known on the internalization mechanism, intracellular localization and trafficking of TCTP-PTD in the context of comparison with TAT-PTD, is reviewed in the following sections.

### 4.1. Cell surface binding, the first step in the entry of a TCTP-PTD into cells

Binding of the PTD to cell surfaces is the first step for cell entry of a PTD into cells. Generally, each cell type has its own unique lipid and glycoprotein components and endocytic machinery, which can influence the interactions of peptides at the initial stage of internalization. Thus, the translocation mechanism can vary depending on the cellular components and specific experimental conditions and the mechanisms in play for each PTD, which can vary accordingly.

An initial electrostatic association of PTDs with cell membrane components such as glycoproteins, induces the reconstruction of the cytoskeletal network (Duchardt et al., 2007; Ziegler, 2008) and facilitates the energy-dependent internalization of PTDs. For cationic PTDs, electrostatic association of basic residues in PTD with a charged phospholipid can mediate the initial step of internalization. Bidentate guanidinium group found in Arg has been shown to be essential for cellular uptake as it avidly binds to sulfated glycans (Lönn & Dowdy, 2015). Additionally, the hydrogen bonding between the Arg guanidino group and phosphates, carboxylates, and sulfates may enhance the hydrophobicity of the peptide, which in turn assists the interaction of PTD with the cell membrane (Rothbard et al., 2004). Lipids and glycosaminoglycan (GAGs) play important roles in the binding of TAT-PTD via hydrogen bonding and specific interactions (Zhang et al., 2012).

The uptake of TCTP-PTD is not dependent on heparan sulfate while that of TAT-PTD requires heparan sulfate proteoglycans for transduction (Kim et al., 2011b). Rather, cellular uptake of TCTP-PTD is sensitive to cholesterol depletion (Kim et al., 2015). The cholesterol-dependent endocytic pathway of TCTP-PTD suggests that cholesterol is a possible interacting molecule required for TCTP-PTD internalization (Kim et al., 2015).

### 4.2. Internalization of the TCTP-PTD

Endocytosis is largely classified as a two part process that includes phagocytosis and pinocytosis. Pinocytosis, the uptake of fluid and solute by the cell, occurs in all types of cells through four pathways including (i) clathrin-mediated, (ii) caveolae-mediated, (iii) clathrin, caveolae-independent pathway, and (iv) macropinocytosis (Conner & Schmid, 2003). Clathrin-mediated endocytosis (CME) is a clathrin/dynamin-dependent, receptor-mediated process and this pathway is reported to operate in the cases of TAT-PTD, oligoarginine, and anionic PTDs (Ruseska & Zimmer, 2020). Binding of ligand to a specific receptor on the cell membrane induces the assembly of clathrins in a polyhedral lattice, followed by the invagination of clathrin-coated membrane surface (Ruseska & Zimmer, 2020). Caveolae-mediated endocytosis (CvME) is mediated by the formation of caveolae, a highly hydrophobic membrane domain rich in sphingolipid and cholesterol (Ruseska & Zimmer, 2020) and is reported to occur with TAT fusion protein, transportan and proline-rich PTDs. Actin cytoskeleton and cholesterol are the essential elements for caveolae formation and dynamin, a multidomain GTPase, constricts the neck of caveolae for enabling its release (Ruseska & Zimmer, 2020).

The lipid raft is a microdomain of the plasma membrane constituted by the interaction of sphingolipid and sterol, which facilitates the internalization of certain macromolecules by clathrin- and caveolae-independent endocytic pathways (Ruseska & Zimmer, 2020). This coat-free pathway can be dynamin-dependent or -independent and is reported in the uptake of PTDs such as azurin fragments and transportan (Ruseska & Zimmer, 2020). Macropinocytosis is a lipid raft-dependent endocytic process, that involves actin-driven plasma membrane protrusion that induces the uptake of extracellular fluids (Ruseska & Zimmer, 2020). Ingestion of extracellular fluids by the membrane ruffles occurs by the action of the actin cytoskeleton (Swanson, 2008; van den Berg & Dowdy, 2011). TAT-protein conjugates and Poly-Arg internalize cells via macropinocytosis (Ruseska & Zimmer, 2020).

It has been reported that TAT-PTD or TAT-PTD-cargo into cells involved direct translocation or internalization through different endocytic pathways (Zhang et al., 2012). It is to note that the speed of uptake largely relies on the labeling tags (Joliot & Prochiantz, 2004) and that the specific uptake pathway for a PTD depends heavily on the cargo (Ruseska & Zimmer, 2020). For example, unconjugated TAT-PTD was reported to be internalized via clathrin-mediated endocytosis (Richard et al., 2005) whereas macropinocytosis (Wadia et al., 2004) and caveolae-mediated endocytosis have been reported to operate for TAT-protein conjugates (Ferrari et al., 2003; Fittipaldi et al., 2003).

It has been shown that TCTP-PTD internalizes the membrane via an energy-dependent endocytosis (Kim et al., 2011b). Our initial study using HeLa cells showed that cellular uptake of TAMRA-labeled TCTP-PTD does not involve the clathrin-mediated endocytosis, but it occurs mostly through lipid raft-mediated endocytosis and partially by macropinocytosis (Kim et al., 2011b).

Our research group performed in-depth verification of the translocation mechanism of TCTP-PTD in A549 lung carcinoma cells (Kim et al., 2015). Consistent with previous findings in HeLa cells (Kim et al., 2011b), TCTP-PTD conjugated with either TAMRA or FITC and TCTP-PTD-GFP fusion protein penetrates into A549 cells via cholesterol-dependent lipid raft/caveolae-mediated endocytosis (Kim et al., 2015). Dynamin and actin polymerization facilitates vesiculation during caveolae-mediated endocytosis, and the endocytic internalization of TCTP-PTD was shown to be dynamin, actin, and microtubule-dependent (Kim et al., 2015). Macropinocytosis that can occur by dynamin-mediated vesiculation with ruffling of biological membranes through actin rearrangement, also is partly involved in the internalization of TAMRA-TCTP-PTD (Kim et al., 2015). As shown with TCTP-PTD internalization, identical PTDs simultaneously utilize several distinctive internalizing pathways.

### 4.3. Intracellular trafficking of TCTP-PTD

After the caveolae-mediated endocytosis, TCTP-PTD is confined to the endocytic vesicles, caveosome, formed by caveolae-driven mechanisms in A549 cells (Kim et al., 2015). GFP-tagged TCTP-PTD showed localization in the caveolin-1-containing lipid-raft (Kim et al., 2015). Then the caveosome containing TCTP-PTD-GFP is transported to the cytoplasm and partly to the cytoskeletal matrix, possibly passing through the ER (Kim et al., 2015), possibly following the retrograde transport route as shown with other PTDs (Patel et al., 2007). In addition, cellular uptake of TCTP-PTD partly involves macropinocytosis (Kim et al., 2011b, 2015) and the fate of macropinosomes may vary according to the cell (Patel et al., 2007). Following the macropinocytosis-mediated transport, macropinosome-laden TCTP-PTD appears to be transported into the cytoplasmic compartment via an as yet unknown mechanism of endosomal exit (Kim et al., 2015).

Although TCTP-PTD penetrates partly via lipid raft-dependent endocytosis and partly via macropinocytosis in both A549 and HeLa cells (Kim et al., 2011b, 2015), the sensitivity to nystatin, an inhibitor of caveolae-mediated endocytosis, differed between two cell lines (Kim et al., 2015). TCTP-PTD transduction in HeLa cells was less affected by nystatin than that in A549 cells (Kim et al., 2015). The higher expression of caveolin-1 in A549 than in HeLa cells supports the cell type-dependent internalization mechanism, which can be influenced by the specific composition of endocytic machinery such as caveolin-1 and lipid rafts (Kim et al., 2015).

The subcellular localization and the fate of PTD-cargo complex is important for the proper and desirable action of cargos. All the endocytic pathways used involve the formation of endosome, followed by lysosomal degradation unless they escape from endosome (van den Berg & Dowdy, 2011). Endosomal escape is essential for the action of cargos in the cytoplasm or nucleus (Zhang et al., 2012). Our earlier study showed that TCTP-PTD itself may escape from lysosomes in contrast to TAT-PTD that needs an endolysomotropic agent, chloroquine, for facilitating its translocation (Kim et al., 2011b). It is speculated that the degree of hydrophobicity of TCTP-PTD might contribute to the process of endosomal escape from endosomes (Kim et al., 2015) and this needs to be clarified in further studies.

In contrast to TAT-PTD that localizes in the nucleus after uptake because of its nuclear localization signal (NLS) (Chauhan et al., 2007), TCTP-PTD showed dispersed distribution in the cytoplasmic compartment but not in the nucleus and lysosomes in HeLa (Kim et al., 2011b). The localization of TCTP-PTD-GFP also showed a non-nuclear distribution in A549 cells (Kim et al., 2015) because TCTP-PTD retains its hydrophobic nature devoid of the functional NLS. Theoretically, non-nuclear localization of TCTP-PTD enables it to avoid the undesirable ionic interactions with nucleic acids, thereby reducing the potential risk of disturbance of normal genetic expression. Because TCTP-PTD does not localize in nucleus and lysosome, it may mediate the delivery of therapeutic cargos into the cytoplasm more efficiently than polycationic PTDs, by avoiding both nuclear accumulation and lysosomal degradation of the PTD-cargo complex.

To sum up, after uptake by lipid raft/caveolae-mediated endocytosis and partly by macropinocytosis that is clathrin-independent, dynamin/cholesterol-dependent pathways, TCTP-PTD in caveolae appears to escape from the endosomal compartment, possibly followed by transfer through ER (Kim et al., 2015). Then it moves to the cytoplasmic part and cytoskeleton containing actin and tubulin, as schematically illustrated in our previous report (Kim et al., 2015). It has been reported that TAT protein is secreted from the TAT-expressing cells and then it reenters the cells by means of its PTD domain. Moreover, TAT protein or TAT-PTD can move from one to another cell, in a mode of transcellular transport (Chauhan et al., 2007). The specific mechanisms responsible for the cytosolic release and the cell-to-cell translocation of TCTP-PTD are of interest but remain elusive.

## 5. Usefulness of TCTP-PTD for drug delivery

**Figure 2.**
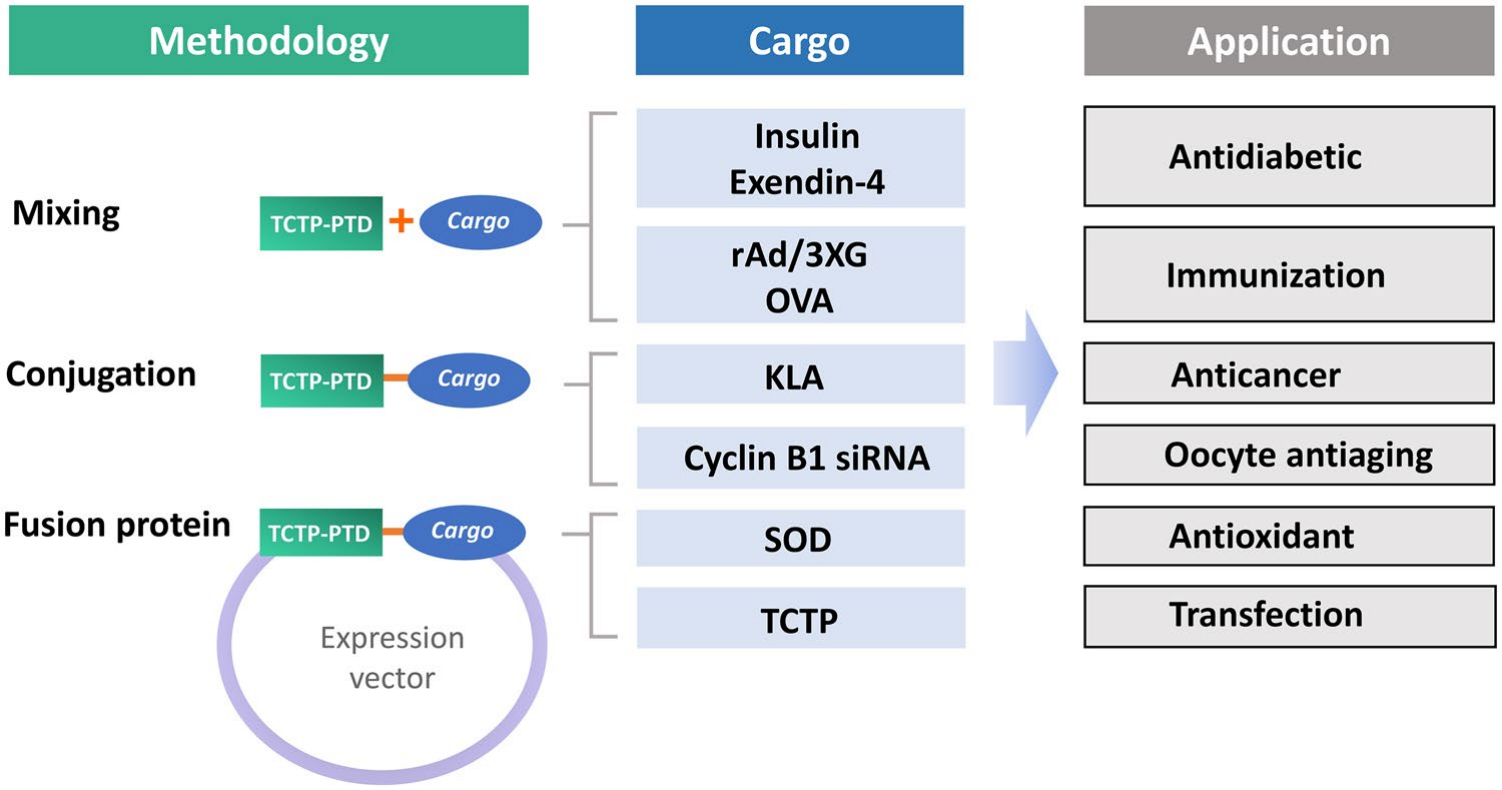
Studies using the TCTP-PTD with various cargos. TCTP-PTD or its variants were complexed with various cargos by simple mixing (for insulin, exendin-4, ovalbumin, and rAd/3XG), covalent conjugation (for KLA and cyclin B1 siRNA), or fusion protein (SOD and TCTP). TCTP-PTD and their effectiveness in the delivery of therapeutics for diabetes, cancer and brain damage, and of antigens for immunization were studied.

PTDs are believed to be preferrable for use in the delivery of macromolecular therapeutics because of their relative high efficiency and low toxicity in the delivery of proteins, nucleic acids, phosphopeptides and peptide nucleic acids (PNAs) (Joliot & Prochiantz, 2004). Many PTDs are currently under the clinical trials for their usefulness in the delivery of specific proteins and peptides (van den Berg & Dowdy, 2011). It has been studied the potential of TCTP-PTD and its variants as carriers of various cargos such as peptides (KLA, insulin, and exendin-4), proteins (β-Gal, SOD, and TCTP), antigen (ovalbumin and rAd/3XG), and nucleic acids (cyclin B1 siRNA) in vivo, and demonstrated its potential usefulness in drug delivery (Figure 2). Because TCTP-PTD is derived from a human protein, it can serve as a safe carrier for the bioactive molecules in both fused and covalently/noncovalently linked forms.

### 5.1. TCTP-PTD-cargo complexes applied in drug delivery

There are three strategies for preparing TCTP-PTD-cargo complexes used by our group and others (Table 1). First, covalent conjugation of TCTP-PTD with cargo was accomplished employing various chemical links. Second, protein cargos can be expressed with TCTP-PTD in a recombinant protein where the cargo can be located at the N- or C-terminus of TCTP-PTD with an appropriate type of linker. The third strategy is physical mixing of TCTP-PTD with cargo, allowing them to form complexes by intermolecular associations such as hydrophobic or electrostatic interactions (Gros et al., 2006).

**Table 1.** Examples of use of TCTP-PTD and its variants in the delivery of various cargos.

Name	Sequence	pI^a^	Cargo	Method	Reference
TCTP-PTD	MIIYRDLISH	6.50	rAd/3XG	simple mixing	(Kim et al., 2011b)
			KLA	conjugation	(Kim et al., 2011a)
			Cyclin B1 siRNA	conjugation	(Jeon et al., 2019)
			SOD	fusion protein	(Lee et al., 2011)
			TCTP	fusion protein	(Maeng et al., 2013)
TCTP-PTD13	MIIFRALISHKK	11.17	insulin	simple mixing	(Bae & Lee, 2013)
			OVA	simple mixing	(Bae et al., 2016)
TCTP-PTD13M2	MIIFRLLASHKK	11.17	Exendin-4	simple mixing	(Bae et al., 2018a)
			insulin	simple mixing	(Bae et al., 2018b)

^a^The theoretical isoelectric point was calculated using the tool of the ExPasy server.

In general, the nature of the link between the cargo and the PTD is important. The linkages can be permanent if they are formed in a fusion protein, or not permanent such as when a disulfide bond can induce the cytoplasmic release of the cargo by the action of cytoplasmic glutathione (Joliot & Prochiantz, 2004). Furthermore, the observed differential intracellular localization of TCTP-PTD and TAT-PTD (Kim et al., 2011b) indicates that selection of PTD can determine intracellular targeting. For example, a cargo having high affinity for the cytoplasmic components may deliver the cargo within the cytoplasmic compartment.

It is known that PTD-cargo conjugates made via covalent links and fusion protein have the potential risk of interfering with the original structure, function, and transepithelial penetration of therapeutic cargos (Kristensen et al., 2015). In this context, the simple mixing method that forms a complex via noncovalent interactions may be preferred. This method has a lower chance of interfering with the unique structure of cargos than fusion or chemical crosslinking method. The strategy of physically mixing PTD with cargo provides an easy way for adjusting the molar ratio between PTD and cargo and simple mixing methods can help the optimized interaction compared to chemically linked or conjugated forms.

### 5.2. Examples of use of TCTP-PTD in drug delivery

#### 5.2.1. Intranasal delivery of antidiabetics-Insulin

Intranasal drug administration is a noninvasive approach that enables rapid absorption of the drug through nasal epithelium, bypassing the hepatic first-pass metabolism (Song et al., 2004). Its major limitation is the low membrane permeability of this route to macromolecular drugs because the rate of absorption of a drug is highly dependent on its molecular size and hydrophilicity (Davis & Illum, 2003). Several PTDs, including TAT-PTD, penetratin, and Poly-Arg, have been employed for insulin delivery, including co-administration or conjugation of PTD with insulin (Liang & Yang, 2005; Morishita et al., 2007; Khafagy et al., 2009).

Our research group examined the potential of intranasal insulin delivery by TCTP-PTD (Bae & Lee, 2013) and other TCTP-PTDs such as TCTP-PTD3 (MIIFRDLISH), TCTP-PTD8 (MIIYRIAASHKK), and TCTP-PTD13 (MIIFRALISHKK). TCTP-PTD13 was found to be the best for intranasal insulin delivery as it decreased blood glucose level in both normal and streptozocin-induced diabetic mouse models without mucosal toxicity. The optimal molar ratio of insulin:TCTP-PTD13 was found to be 1:2. The relative pharmacological bioavailability (F%) following intranasal administration of TCTP-PTD 13/insulin was 21.3% compared with subcutaneous insulin injection (Bae & Lee, 2013).

It has been reported that the molecular binding of PTD with drugs is crucial in nasal drug delivery (Khafagy et al., 2010). Noncovalent intermolecular binding of TCTP-PTD with insulin was suggested to mediate the penetration of insulin into the nasal epithelium. TCTP-PTD13 was found to be distributed in the submucosal region of mice after intranasal administration, suggesting mucosal penetration of PTD (Bae & Lee, 2013).

We designed other modified variants of TCTP-PTD13 (MIIFRALISHKK) to further enhance the efficiency of nasal insulin delivery (Bae et al., 2018a). Because 1-MIIFR-5 and 9-SHKK-12 of TCTP-PTD13 have essential residues for efficiency and water solubility, 6-ALI-8 residues were subjected to modification and tested. Based on our previous results with TCTP-PTD 8 (MIIYRIAASHKK) (Bae & Lee, 2013), the substitution of 6-ALI-8 to 6-IAA-8 was ruled out because it did not enhance the efficiency.

To increase the hydrophobic nature of the PTD, residue ^6 ^A was substituted by ^6 ^L (TCTP-PTD13M1, MIIFRLLISHKK). However, TCTP-PTD13M1 did not improve the efficiency and solubility, possibly due to the effect of steric hindrance from the side chain of Leu. This led to the design of the variant TCTP-PTDM2 (MIIFRLLASHKK) that contains both A6L and I8A substitutions in TCTP-PTD13 to decrease any potential steric hindrance and to retain its hydrophobic nature (Bae et al., 2018a).

Pharmacokinetic studies showed that TCTP-PTD13M2 promoted an enhanced intranasal insulin delivery than TCTP-PTD13 and TCTP-PTD13M1. The relative bioavailability of nasally administered TCTP-PTDM2 in normal rats was 37.1% relative to subcutaneous injection, which was 1.68 times higher that of insulin/TCTP-PTD13 intranasal administration (Bae et al., 2018a). In alloxan-induced diabetic rats, nasal TCTP-PTD13M2/insulin significantly increased the hypoglycemic effect of TCTP-PTD13 without mucosal toxicity (Bae et al., 2018a). Substitution of ^8^Ile to ^8^Ala of TCTP-PTD13M1 improved the efficiency of delivery but also reduced the precipitation of complexes.

When we changed the stereochemistry of TCTP-PTD13 and TCTP-PTD13M2, respectively, from the L- to the D-forms, the D-forms did not exhibit enhanced efficiency of intranasal delivery. In addition, a higher degree of complex formation was found by turbidity analysis (Bae et al., 2018a). However, in a study of intranasal delivery of ovalbumin, the D-form of TCTP-PTD13 showed an enhanced efficiency than the L-form (Bae et al., 2016), suggesting that the nature of the cargo can affect PTD transduction.

#### 5.2.2. Intranasal delivery of antidiabetics-Exendin-4

Exendin-4 is an incretin-mimetic peptide comprising 39 amino acids used for treating type 2 diabetics as an agonist for glucagon-like peptde-1 (GLP-1) (Yap & Misuan, 2019). Exendin-4 has a longer half-life than GLP-1 because of its resistance to enzymatic degradation (Yap & Misuan, 2019). TCTP-PTD/exendin-4 complexes were formed during the simple mixing method that was employed for the intranasal insulin delivery. In addition to the variants including TCTP-PTD13, 13M1, and 13M2, TCTP-PTD13M3 (MIIFRLLAYHKK), other variants developed by S9Y substitution of TCTP-PTD13M2, were studied in attempts to enhance their solubility due to the addition of the hydroxy group contained in non-polar Tyr residue.

The plasma exendin-4 levels following intranasal administration were observed to be higher with the usage of the following PTDs in the following order: TCTP-PTD13M2, 13M3, 13M1, and 13. Thus, TCTP-PTD13M2 was found to be the best PTD for exendin-4 delivery (Bae et al., 2018a). Intranasally delivered TCTP-PTD13M2/exendin-4 reduced blood glucose levels by 43.3% compared with that of intranasal exendin-4 and by 18.6% compared with that of intranasally delivered TCTP-PTD13/exendin-4 in *db/db* mice (Bae et al., 2018a).

To delineate the potential impact of the method for linking and the location of PTD in complexes, the TCTP-PTD-exendin-4 complexes were formed by the conjugation via a peptide bond. Exendin-4 was attached to the C- or N-terminus of TCTP-PTD13M2 via GGG (Gly-Gly-Gly) linker, designated as C-M2-Exendin-4 and N-M2-Exendin-4, respectively. The GGG linker was introduced to reduce the possible steric hindrance between PTD and cargo peptides. The hypoglycemic effect of subcutaneously injected C-M2-Exendin-4 and N-M2-Exendin-4 was inferior than the effect of subcutaneously administered exendin-4 in *db/db* mice (Bae et al., 2018a).

Intranasal administration of C-M2-Exendin-4 had little hypoglycemic effect, whereas the mixture of TCTP-PTD13M2/Exendin-4 showed significantly greater effect in *db/db* mice (Bae et al., 2018a). An unknown mechanism may be involved in the differential efficiencies between conjugated and mixed complexes, such as the instability of the C-M2-Exendin-4 under the experimental conditions. Overall, simple mixing methods may be preferrable for TCTP-PTD-mediated intranasal administration of antidiabetic peptides.

### 5.3. Intranasal delivery of antigens

#### 5.3.1. Adenoviral gene delivery (rAd/3XG)

Nasal vaccination is a noninvasive route that induces both systemic and mucosal immunity (An et al., 2021). Intranasal vaccine delivery provides an easily accessible pathway to the immune system. The combined effect of the charge and size of the antigen is the key to gain optimal immunological effect (Yusuf & Kett, 2017). To facilitate the intranasal antigen delivery for immunization, novel approaches, including the use of a PTD, such as TAT-PTD and penetratin, have been used (Muto et al., 2016; Yin et al., 2020). TCTP-PTD and its variants showed promise for use in intranasal vaccination in our studies. The simple mixing method was applied for the PTD/cargo complex formation (Kim et al., 2011b; Bae et al., 2016).

The attached glycoprotein (G) that targets the ciliated cells of the airway and the fusion glycoprotein (F) on the surface of the respiratory syncytial virus (RSV) are the two major glycoproteins that prime the initial phase of the infection (Levine et al., 1987; McLellan et al., 2013). Intranasal immunization of rAd/3XG, the adenovirus encoding the triple repeat of the G glycoprotein fragment of RSV, induces the immunity against RSV (Yu et al., 2008). Intranasal administration of the complex formed by preincubation of TCTP-PTD with rAd/3XG, efficiently induced the humoral responses in mice, as shown by the increase in both systemic anti-RSV IgG and RSV-specific secretory IgA response (Kim et al., 2011b). TCTP-PTD enhanced the adenovirus-mediated gene expression by improving antigen-specific mucosal and humoral immune responses in vivo.

#### 5.3.2. Ovalbumin (OVA)

Another study using TCTP-PTD in intranasal antigen delivery was conducted with ovalbumin (OVA). OVA, is the major protein found in egg-white (Yu et al., 2008). OVA-sensitized and challenged mice are commonly used as an allergy model (Kim et al., 2019). L- and D-forms of TCTP-PTD13 (MIIFRALISHKK) were used for intranasal OVA delivery and PTD/OVA complexes were formed by a simple mixing method (Bae et al., 2016).

Intranasal administration of D-TCTP-PTD13/OVA complex showed higher efficiency in inducing the production of plasma IgE and secretory IgA than nasally administered OVA, L-TCTP-PTD13/OVA, and i.m. administered OVA in mice. Synthetic D-form peptides are more resistant than naturally-occurring L-form peptides to enzymatic degradation. The enhanced stability of the peptides may contribute to an enhanced internalization across the mucosal epithelium. D-TCTP-PTD/OVA induced a Th2-biased immune response as indicated by the dominant IgG1 subclass in plasma IgG, as also observed in i.m. administration of OVA (Bae et al., 2016).

To induce Th1-immune response, a CpG oligonucleotide (CpG) that has Th1-promoting ability, was used as an immunostimulatory adjuvant. Intranasal administration of D-TCTP-PTD13/OVA/CpG mixture complex in mice not only enhanced the immunization efficiency but also modulated the immune responses toward Th1, by adjusting IgG1 and IgG2a profiles, suggesting the value of TCTP-PTD13 in the intranasal antigen delivery for vaccination (Bae et al., 2016). D-TCTP-PTD13 showed molecular binding with CpG, possibly through hydrophobic and/or electrostatic interactions (Bae et al., 2016).

### 5.4. Conjugation of other cargos and delivery studies with TCTP-PTD

#### 5.4.1. KLA

The proapoptotic peptide KLAKLAKKLAKLAK (KLA) is a cationic amphipathic sequence that can disrupt the mitochondrial membrane that in turn facilitating programmed cell death (Ellerby et al., 1999; Foillard et al., 2008). KLA is generally nontoxic to eukaryotic cells because of its impermeability across the plasma membrane. It has been reported that the fusion of KLA with cationic PTDs, including TAT-PTD induced the toxicity of KLA because of PTD-driven KLA internalization (Law et al., 2006; Kwon et al., 2008).

Our previous study used FITC-labeled TCTP-KLA (TCTP-PTD-conjugated KLA) and TAT-KLA (TAT-PTD-conjugated KLA) containing GG linker (Kim et al., 2011a). TAT-KLA showed superior transduction efficiency than TCTP-KLA in several cancer cell lines, including A549, HeLa, HepG2, AGS, ACHN, and SK-Hep1 (Kim et al., 2011a). TCTP-KLA showed cell death-inducing activities with IC_50_ of 7-10 μmol/L in four cancer cell lines, while TAT-KLA had IC_50_ of 2-10 μmol/L among all cells. TCTP-KLA exhibited better efficacy in ACHN cells (stomach carcinoma) whereas KLA-PTD showed the best activity in AGS (renal carcinoma) cells (Kim et al., 2011a). These results indicate that the ability for cellular penetration does not correlate with intracellular biological activity. In addition to the cell translocation ability, the physical and biological nature of PTD-cargo, and the cell-specific factors are the determinants of biological effects of cargos. TCTP-PTD-aided delivery of KLA in vitro was confirmed to induce apoptosis by activating apoptotic pathways involving caspase-3 and PARP (Kim et al., 2011a).

Stability assays of TCTP-KLA using mouse serum showed that about 60% of TCTP-KLA remained in the serum for 12 h, suggesting favorable stability in vivo (Kim et al., 2011a). In vivo efficacy analysis using a xenograft mouse model of human lung carcinoma indicated that injection of TCTP-KLA into xenografts established in nude mice regresses the tumor growth more efficiently than that of TAT-KLA (Kim et al., 2011a). This implies that the hydrophobic nature of TCTP-PTD enables it to traverse efficiently into tissues in vivo.

#### 5.4.2. Cyclin B1 siRNA

Although conjugation of TAT-PTD with siRNA enhanced the cellular delivery of oligonucleotide (Chiu et al., 2004), the possible interference on gene expression by TAT-PTD - is of consideration (Wender et al., 2000). The intracellular behavior of TCTP-PTD is favorable for the cytoplasmic delivery of siRNA by avoiding the nuclear localization and endosomal entrapment.

Delivery of siRNA using TCTP-PTD across zona pellucida (ZP) in mouse oocytes was reported by Jeon *et al* (Jeon et al., 2019). The zona pellucida, a glycoprotein matrix that surrounds the biological membranes of embryos and oocytes, limits the permeation of exogenous molecules into mammalian embryos and oocytes during prolonged in vitro culture (Wassarman, 2008; Gupta et al., 2012). This group previously found that overexpression of TCTP can attenuate the time-dependent quality deterioration and apoptosis of oocytes (Jeon et al., 2017). Here, the exogenous TCTP, a form of recombinant TCTP-mCherry, also relieved the quality decline of oocytes when added to culture media, which in turn improved the fertilization capacity and subsequent development of early embryos (Jeon et al., 2019). TCTP-mCherry protein devoid of PTD showed impermeability, confirming the PTD-dependent translocation of TCTP.

Exogenous TCTP can translocate into oocytes across the ZP by the action of the PTD and the internalized TCTP eventually retains its physiological characteristics. Once internalized, TCTP-mCherry was found to be localized in the cortex and extensively distributed within the cytoplasm (Jeon et al., 2019). Furthermore, this group linked TCTP-PTD with cyclin B1 siRNA via a thiol-maleimide coupling. This complex was translocated into oocyte cytoplasm, which eventually induced the downregulation of cyclin B1 (Jeon et al., 2019). TCTP itself can penetrate oocyte and is a noninvasive and valuable vehicle for siRNA delivery into the mouse oocyte.

#### 5.4.3. Superoxide dismutase (SOD)

Superoxide radical is produced during metabolism of oxygen in plasma membrane and mitochondria. High levels of reactive oxygen species (ROS) cause oxidative stress and cell cytotoxicity (Mondola et al., 2016). Superoxide dismutases (SODs) belong to the isoenzyme family that scavenges superoxide anions (Mondola et al., 2016). Cu,Zn-SOD (SOD1) is highly expressed in cytosol and partly in the mitochondrial matrix (Mondola et al., 2016). Application of SOD as neuroprotective antioxidants is limited because of the poor permeability of SOD into the brain. Several PTDs were considered as potential vehicles for the delivery of Cu,Zn-SOD across the blood brain barrier (BBB) (Asoh and Ohta, 2008).

TCTP-SOD and TAT-SOD recombinant proteins that have TCTP-PTD or TAT-PTD moiety at the N-terminus of Cu,Zn-SOD were designed for the application in neuronal damage (Lee et al., 2011). TCTP-SOD and TAT-SOD showed cellular transduction into HeLa, SH-SY5Y, and HaCaT cells after incubation for 6 or 24 h (Lee et al., 2011). Cellular uptake of TCTP-SOD tends to require longer incubation time compared to TAT-SOD (Lee et al., 2011).

Treatment with paraquat induces intracellular generation of superoxide anion and subsequent cell damage by oxidative stress (McCarthy et al., 2004). TAT-SOD showed better efficiency than TCTP-SOD although both complexes protected the cells from the paraquat-induced cell cytotoxicity in SH-SY5Y cells (Lee et al., 2011). Following intraperitoneal injection of TCTP-SOD and TAT-SOD in mice, both were delivered to the hippocampal region of the brain and protected brain cells from kainic acid-induced neuronal damage. Kainic acid, a cyclic analog of L-glutamate, is a potent neuroexcitatory agent that binds to glutamate receptor and induces an influx of calcium into cytoplasm, followed by the activation of free radical-generating enzymes (Coyle & Puttfarcken, 1993), eventually resulting in brain damage. Of note, TCTP-SOD showed more extensive distribution in the hippocampal region and enhanced protection from neuronal deaththan that of TAT-SOD (Lee et al., 2011), indicating an efficient translocating ability of TCTP-PTD through the blood brain barrier (BBB).

Interestingly, the translocation of TCTP-PTD across BBB also appears to depend on the type of cargo because TCTP-β-Gal fusion protein exhibited negligible transduction of β-Gal into the mouse brain (Kim et al., 2011b). The higher in vivo efficacy of TCTP-PTD than TAT-PTD in the delivery of SOD and KLA indicates the unique behavior of hydrophobic TCTP-PTD.

#### 5.4.4. TCTP

Full-length TCTP retaining N-terminal 1-10 amino acids, *per se* can internalize into cells, whereas N11TCTP (TCTP11-172) devoid of the PTD region cannot translocate the cells. To increase the efficiency of exogenous TCTP delivery using its PTD, GFP-tagged TCTP fusion proteins with one or more TCTP-PTDs at the N and/or C-terminus of TCTP-GFP were constructed and expressed for fusion proteins (Maeng et al., 2013). The potential impact of location and the number of PTDs in TCTP delivery was explored in A549 cells. The presence of one or two TCTP-PTD at its N-terminus showed enhanced transduction than either the C-terminal PTD or PTD located both at N- and C-termini (Maeng et al., 2013). Exogenous TCTP internalizes into cells and it retains its biological functions that is the activation of the ERK and PI3K pathway.

Fusion of TCTP-PTD at N-terminal of full-length TCTP exhibited comparable efficiency compared to TCTP (Maeng et al., 2013). Tandem addition of TCTP-PTD at N-terminus of full-length TCTP did not enhance transduction whereas presence of TCTP-PTD in C-terminal decreased the internalization of TCTP. Taken together, the exposure of N-terminal TCTP-PTD appears necessary for protein transduction and C-terminal PTD interferes with the internalization of TCTP (Maeng et al., 2013). Based on the anti-parallel alignment between N- and C-terminal β-strands of TCTP in the NMR structure of human TCTP (Feng et al., 2007), the addition of PTD at C-terminus may negatively affect the exposure of N-terminal TCTP-PTD (Figure 1).

## 6. Physiological significance of TCTP-PTD

PTDs derived from HIV-1 TAT transactivator and the homeodomain of *Drosophila* transcription factor (Frankel & Pabo, 1988; Green & Loewenstein, 1988; Joliot et al., 1991) are suggested to reflect their in vivo physiological functions (Joliot & Prochiantz, 2004). Antp is a homeoprotein that is a transcription factor responsible for morphogenesis during embryogenesis of the *Drosophila*. After cellular uptake, homeodomain acts as a messenger protein by regulating the transcription and translation of specific genes (Prochiantz, 2000; Prochiantz & Joliot, 2003). Some homeodomains are suggested to be involved in their intercellular trafficking through secretion and internalization of the homeodomain. This indicates the role of homeoprotein in providing the signals for internalization and secretion (Dom et al., 2003). TAT protein derived from the HIV-1 can translocate into cells to modulate the transcription of genes (Frankel & Pabo, 1988; Green & Loewenstein, 1988). TAT expressed at high level can be secreted from the cells and reenter bystander cells but not transcellularly (Chauhan et al., 2007).

Based on the observation that the translocating ability of PTDs correlates with that of their full-length proteins, the cellular transduction of PTDs may contribute to the physiological status of the protein in cells. As a part of the physiological process, some full-length proteins containing PTD can be secreted, although the exact mechanism and behavior have not been clearly delineated. These strongly indicate the paracrine function of PTD-containing specific proteins.

In this context, the existence and conditional truncation of the PTD domain in N-terminus of TCTP suggest a potential regulation mechanism for TCTP mediated by TCTP-PTD between intracellular and extracellular milieu. Expression of TCTP is highly regulated and its dysregulation is implicated in pathological conditions such as cardiovascular and metabolic diseases, cancer, allergy, and immune disorders (Bommer & Kawakami, 2021). TCTP can be exported out of cells via TSAP6-mediated ER/Golgi-independent or nonclassical pathway (Amzallag et al., 2004) to promote histamine-releasing activities. In this regard, TCTP-PTD is thought to act as a signal peptide that confines PTD-deleted (thus non-penetrable) TCTP in the extracellular compartment where it acts as a histamine-releasing factor.

**Figure 3.**
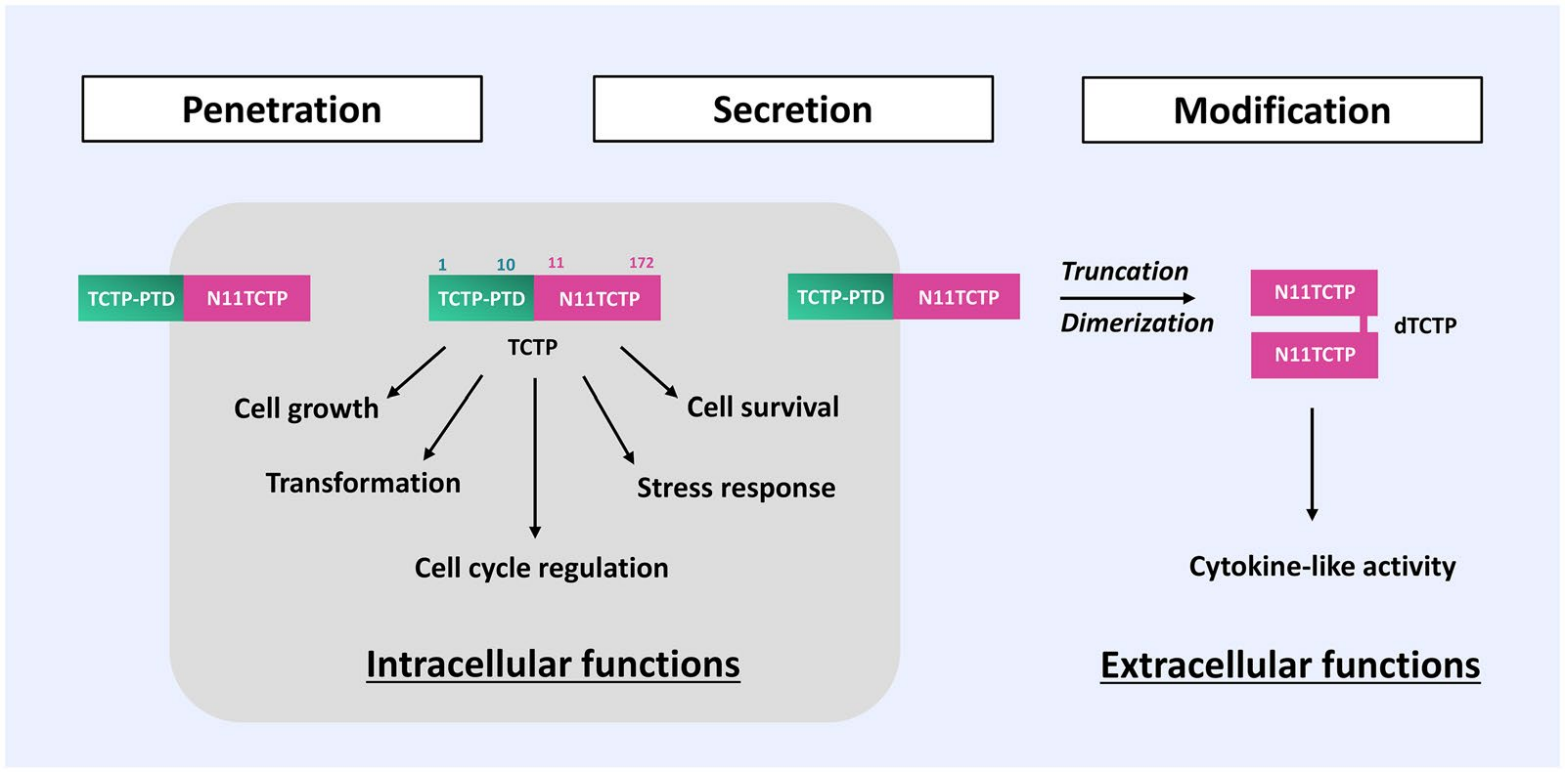
Role of TCTP-PTD in the regulation of intracellular and extracellular actions. TCTP itself can translocate into cells and retains its intracellular activity. Following secretion of TCTP from cells via a non-classical pathway (Amzallag et al., 2004), TCTP is subjected to proteolytic cleavage, resulting in the truncated product TCTP-PTD (1-10), liberating N11TCTP (11-172). N11TCTP is then dimerized under the specific pathologic conditions to form the histamine-releasing factor (HRF), the critical moiety responsible for the extracellular functions of TCTP (Kim et al., 2013). TCTP-PTD-truncated forms, such as N11-TCTP and dTCTP (HRF) cannot cross the plasma membrane, indicating the TCTP-PTD’s role in the plethora of TCTP functions in and out of the cell.

Extracellularly secreted TCTP can undergo a truncation of N-terminal TCTP-PTD under specific conditions and the truncated TCTP (11-172) can be dimerized, acquiring its cytokine-like activities (Kim et al., 2013; Figure 3). As shown in Figure 1, the truncation of N-terminal TCTP-PTD may contribute to the structural modification that exposes the C-terminal domain where the dimerization of TCTP via intermolecular disulfide bond occurs. This concept requires further clarification and verification (Kim et al. 2009, 2013). The physiological significance of TCTP-PTD in the regulation of intracellular and extracellular functions still awaits robust validation. This may help delineate the plethora of TCTP functions in human physiology and pathology.

## 7. Conclusion

Modern drug delivery techniques have been evolved to overcome the delivery challenges using the strategic drug delivery systems, including microneedle patch, microparticle depot, coated microparticle, lipid-base nanoparticles, microencapsulation, and swellable hydrogels (Vargason et al., 2021). In order to transport and release the drugs into the target site or cells in the body, targeted delivery systems are being applied using targeting-ligand conjugates (Vargason et al., 2021). In comparison with recent drug delivery systems, PTD-aided drug delivery might have some limitations in the targeted drug delivery efficiencies because of the molecular characteristics of the peptides and the absence of targeting moieties. However, the emerging strategies on the PTDs grafting with these updated drug delivery systems by conjugating the targeting moiety and nanoparticle carriers (Gessner & Neundorf et al., 2020) shed light on their potential application in advanced and delicate PTD-based drug delivery systems.

Current evidence collectively indicates that naturally-occurring TCTP-PTD has unique characteristics compared to well-known TAT-PTD (Table 2), suggesting it as a new type of carrier for drug delivery. While the human protein-originated Hph-1 (Choi et al., 2006) and lactoferrin-derived PTD (Duchardt et al., 2009) showed lower efficiency than TAT-PTD (Lim et al. 2012), TCTP-PTD exhibited better efficiency than TAT-PTD in the delivery of cargos in vivo. Therefore, TCTP-PTD can be regarded as a potential vehicle for the delivery of macromolecules, such as peptide, protein, and siRNA and for the noninvasive delivery of therapeutics. It is necessary for the research on the strategies for cell-specific targeting of TCTP-PTD and on the optimization of the specific TCTP-PTD-cargo complex to facilitate its application in drug delivery.

**Table 2.** Comparison of TCTP-PTD with TAT-PTD.

	TCTP-PTD	TAT-PTD
Sequence	MIIYRDLISH (TCTP _1-10_)	GRKKRRQRRR (Tat _48-57_)
Class	Hydrophobic PTD	Cationic PTD
Origin	Human TCTP (a housekeeping protein)	HIV-1 Tat (a transactivator of transcription)
Mechanism (Unconjugated)	Lipid-raft/caveolae-mediated endocytosis and pinocytosis (Kim et al. 2011b, 2015)	Clathrin-mediated endocytosis (Richard et al., 2005)
Cell-surface binding (Kim et al., 2011b)	Heparan sulfate-independent uptake	Heparan sulfate-dependent uptake
Localization	Non-nuclear localization (Cytoplasm/Cytoskeleton) (Kim et al. 2011b, 2015)	Nuclear localization (Chauhan et al., 2007)
Kinetics (Kim et al., 2011b)	Relatively efficient at higher concentration in later time points	Relatively efficient at lower concentration in earlier time points

## References

[CIT0001] Amzallag N, Passer BJ, Allanic D, et al. (2004). TSAP6 facilitates the secretion of translationally controlled tumor protein/histamine-releasing factor via a nonclassical pathway. J Biol Chem 279:46104–12. 10.1074/jbc.M404850200 15319436

[CIT0002] An X, Martinez-Paniagua M, Rezvan A, et al. (2021). Single-dose intranasal vaccination elicits systemic and mucosal immunity against SARS-CoV-2. iScience 24:103037. 10.1016/j.isci.2021.103037 34462731 PMC8388188

[CIT0003] Asoh S, Ohta S. (2008). PTD-mediated delivery of anti-cell death proteins/peptides and therapeutic enzymes. Adv Drug Deliv Rev 60:499–516. 10.1016/j.addr.2007.09.011 18093693

[CIT0004] Bae H, Lee K. (2013). On employing a translationally controlled tumor protein-derived protein transduction domain analog for transmucosal delivery of drugs. J Control Release 170:358–64. 10.1016/j.jconrel.2013.06.010 23791976

[CIT0005] Bae H-D, Kim M, Lee J, Lee K. (2018a). Modified translationally controlled tumor protein-derived protein transduction domain enhances nasal delivery of exendin-4 as shown with insulin. Drug Deliv 25:1579–84. 10.1080/10717544.2018.1491653 30044154 PMC6096457

[CIT0006] Bae H-D, Lee J, Jin X-H, Lee K. (2016). Potential of translationally controlled tumor protein-derived protein transduction domains as antigen carriers for nasal vaccine delivery. Mol Pharm 13:3196–205. 10.1021/acs.molpharmaceut.6b00408 27454469

[CIT0007] Bae H-D, Lee J, Jun K-Y, et al. (2018b). Modification of translationally controlled tumor protein-derived protein transduction domain for improved intranasal delivery of insulin. Drug Deliv 25:1025–32. 10.1080/10717544.2018.1464081 29688087 PMC6058520

[CIT0008] Bienert S, Waterhouse A, de Beer TAP, et al. (2017). The SWISS-MODEL repository-new features and functionality. Nucleic Acids Res 45:D313–D319. 10.1093/nar/gkw1132 27899672 PMC5210589

[CIT0009] Bommer U-A, Kawakami T. (2021). Role of TCTP in cell biological and disease processes. Cells 10:2290. 10.3390/cells10092290 34571939 PMC8471051

[CIT0010] Bommer U-A, Thiele B-J. (2004). The translationally controlled tumour protein (TCTP). Int J Biochem Cell Biol 36:379–85. 10.1016/s1357-2725(03)00213-9 14687915

[CIT0011] Chauhan A, Tikoo A, Kapur AK, Singh M. (2007). The taming of the cell penetrating domain of the HIV Tat: myths and realities. J Control Release 117:148–62. 10.1016/j.jconrel.2006.10.031 17196289 PMC1859861

[CIT0012] Chiu Y-L, Ali A, Chu C-Y, et al. (2004). Visualizing a correlation between siRNA localization, cellular uptake, and RNAi in living cells. Chem Biol 11:1165–75. 10.1016/j.chembiol.2004.06.006 15324818

[CIT0013] Choi J-M, Ahn M-H, Chae W-J, et al. (2006). Intranasal delivery of the cytoplasmic domain of CTLA-4 using a novel protein transduction domain prevents allergic inflammation. Nat Med 12:574–9. 10.1038/nm1385 16604087

[CIT0014] Conner SD, Schmid SL. (2003). Regulated portals of entry into the cell. Nature 422:37–44. 10.1038/nature01451 12621426

[CIT0015] Coyle JT, Puttfarcken P. (1993). Oxidative stress, glutamate, and neurodegenerative disorders. Science 262:689–95. 10.1126/science.7901908 7901908

[CIT0016] Davis SS, Illum L. (2003). Absorption enhancers for nasal drug delivery. Clin Pharmacokinet 42:1107–28. 10.2165/00003088-200342130-00003[PMC][14531723] 14531723

[CIT0017] Derossi D, Joliot AH, Chassaing G, Prochiantz A. (1994). The third helix of the Antennapedia homeodomain translocates through biological membranes. J Biol Chem 269:10444–50. 8144628

[CIT0018] Dom G, Shaw-Jackson C, Matis C, et al. (2003). Cellular uptake of Antennapedia Penetratin peptides is a two-step process in which phase transfer precedes a tryptophan-dependent translocation. Nucleic Acids Res 31:556–61. 10.1093/nar/gkg160 12527762 PMC140524

[CIT0019] Drin G, Déméné H, Temsamani J, Brasseur R. (2001). Translocation of the pAntp peptide and its amphipathic analogue AP-2AL. Biochemistry 40:1824–34. 10.1021/bi002019k 11327845

[CIT0020] Duchardt F, Fotin-Mleczek M, Schwarz H, et al. (2007). A comprehensive model for the cellular uptake of cationic cell-penetrating peptides. Traffic 8:848–66. 10.1111/j.1600-0854.2007.00572.x 17587406

[CIT0021] Duchardt F, Ruttekolk IR, Verdurmen WPR, et al. (2009). A cell-penetrating peptide derived from human lactoferrin with conformation-dependent uptake efficiency. J Biol Chem 284:36099–108. 10.1074/jbc.M109.036426 19858187 PMC2794725

[CIT0022] Ellerby HM, Arap W, Ellerby LM, et al. (1999). Anti-cancer activity of targeted pro-apoptotic peptides. Nat Med 5:1032–8. 10.1038/12469 10470080

[CIT0023] Fawell S, Seery J, Daikh Y, et al. (1994). Tat-mediated delivery of heterologous proteins into cells. Proc Natl Acad Sci U S A 91:664–8. 10.1073/pnas.91.2.664 8290579 PMC43009

[CIT0024] Feng Y, Liu D, Yao H, Wang J. (2007). Solution structure and mapping of a very weak calcium-binding site of human translationally controlled tumor protein by NMR. Arch Biochem Biophys 467:48–57. 10.1016/j.abb.2007.08.021 17897616

[CIT0025] Ferrari A, Pellegrini V, Arcangeli C, et al. (2003). Caveolae-mediated internalization of extracellular HIV-1 tat fusion proteins visualized in real time. Mol Ther J Am Soc Gene Ther 8:284–94. 10.1016/s1525-0016(03)00122-9 12907151

[CIT0026] Fittipaldi A, Ferrari A, Zoppé M, et al. (2003). Cell membrane lipid rafts mediate caveolar endocytosis of HIV-1 Tat fusion proteins. J Biol Chem 278:34141–9. 10.1074/jbc.M303045200 12773529

[CIT0027] Foillard S, Jin Z, Garanger E, et al. (2008). Synthesis and biological characterisation of targeted pro-apoptotic peptide. Chembiochem 9:2326–32. 10.1002/cbic.200800327 18712748

[CIT0028] Frankel AD, Pabo CO. (1988). Cellular uptake of the tat protein from human immunodeficiency virus. Cell 55:1189–93. 10.1016/0092-8674(88)90263-2 2849510

[CIT0029] Futaki S, Suzuki T, Ohashi W, et al. (2001). Arginine-rich peptides. An abundant source of membrane-permeable peptides having potential as carriers for intracellular protein delivery. J Biol Chem 276:5836–40. 10.1074/jbc.M007540200 11084031

[CIT0030] Gessner I, Neundorf I. (2020). Nanoparticles modified with cell-penetrating peptides: conjugation mechanisms, physicochemical properties, and application in cancer diagnosis and therapy. IJMS 21:2536. 10.3390/ijms21072536 PMC7177461 32268473

[CIT0031] Goun EA, Pillow TH, Jones LR, et al. (2006). Molecular transporters: synthesis of oligoguanidinium transporters and their application to drug delivery and real-time imaging. Chembiochem 7:1497–515. 10.1002/cbic.200600171 16972294

[CIT0032] Green M, Loewenstein PM. (1988). Autonomous functional domains of chemically synthesized human immunodeficiency virus tat trans-activator protein. Cell 55:1179–88. 10.1016/0092-8674(88)90262-0 2849509

[CIT0033] Gros E, Deshayes S, Morris MC, et al. (2006). A non-covalent peptide-based strategy for protein and peptide nucleic acid transduction. Biochim Biophys Acta 1758:384–93. 10.1016/j.bbamem.2006.02.006 16545342

[CIT0034] Gross B, Gaestel M, Böhm H, Bielka H. (1989). cDNA sequence coding for a translationally controlled human tumor protein. Nucleic Acids Res 17:8367. 10.1093/nar/17.20.8367 2813067 PMC334973

[CIT0035] Gupta SK, Bhandari B, Shrestha A, et al. (2012). Mammalian zona pellucida glycoproteins: structure and function during fertilization. Cell Tissue Res 349:665–78. 10.1007/s00441-011-1319-y 22298023

[CIT0036] Jeon H-J, Bai G-Y, Park Y, et al. (2019). Prevention of quality decline and delivery of siRNA using exogenous TCTP translocation across the zona pellucida in mouse oocytes. Sci Rep 9:18845. 10.1038/s41598-019-55449-4 31827205 PMC6906282

[CIT0037] Jeon H-J, Cui X-S, Guo J, et al. (2017). TCTP regulates spindle assembly during postovulatory aging and prevents deterioration in mouse oocyte quality. Biochim Biophys Acta Mol Cell Res 1864:1328–34. 10.1016/j.bbamcr.2017.05.002 28476647

[CIT0038] Joliot A, Pernelle C, Deagostini-Bazin H, Prochiantz A. (1991). Antennapedia homeobox peptide regulates neural morphogenesis. Proc Natl Acad Sci U S A 88:1864–8. 10.1073/pnas.88.5.1864 1672046 PMC51126

[CIT0039] Joliot A, Prochiantz A. (2004). Transduction peptides: from technology to physiology. Nat Cell Biol 6:189–96. 10.1038/ncb0304-189 15039791

[CIT0040] Khafagy E-S, Morishita M, Isowa K, et al. (2009). Effect of cell-penetrating peptides on the nasal absorption of insulin. J Control Release 133:103–8. 10.1016/j.jconrel.2008.09.076 18930084

[CIT0041] Khafagy E-S, Morishita M, Takayama K. (2010). The role of intermolecular interactions with penetratin and its analogue on the enhancement of absorption of nasal therapeutic peptides. Int J Pharm 388:209–12. 10.1016/j.ijpharm.2009.12.060 20060451

[CIT0042] Kim DI, Song M-K, Lee K. (2019). Comparison of asthma phenotypes in OVA-induced mice challenged via inhaled and intranasal routes. BMC Pulm Med 19:241. 10.1186/s12890-019-1001-9 31823765 PMC6902567

[CIT0043] Kim HY, Kim S, Pyun HJ, et al. (2015). Cellular uptake mechanism of TCTP-PTD in human lung carcinoma cells. Mol Pharm 12:194–203. 10.1021/mp500547f 25423047

[CIT0044] Kim HY, Kim S, Youn H, et al. (2011a). The cell penetrating ability of the proapoptotic peptide, KLAKLAKKLAKLAK fused to the N-terminal protein transduction domain of translationally controlled tumor protein, MIIYRDLISH. Biomaterials 32:5262–8. 10.1016/j.biomaterials.2011.03.074 21565400

[CIT0045] Kim M, Kim M, Kim HY, et al. (2011b). A protein transduction domain located at the NH2-terminus of human translationally controlled tumor protein for delivery of active molecules to cells. Biomaterials 32:222–30. 10.1016/j.biomaterials.2010.08.077 20863558

[CIT0046] Kim M, Maeng J, Jung J, et al. (2011c). Design and evaluation of variants of the protein transduction domain originated from translationally controlled tumor protein. Eur J Pharm Sci 43:25–31. 10.1016/j.ejps.2011.03.007 21440624

[CIT0047] Kim M, Maeng J, Lee K. (2013). Dimerization of TCTP and its clinical implications for allergy. Biochimie 95:659–66. 10.1016/j.biochi.2012.10.007 23104268

[CIT0048] Kim M, Min HJ, Won HY, et al. (2009). Dimerization of translationally controlled tumor protein is essential for its cytokine-like activity. PloS One 4:e6464. 10.1371/journal.pone.0006464 19649253 PMC2715101

[CIT0049] Kristensen M, de Groot AM, Berthelsen J, et al. (2015). Conjugation of cell-penetrating peptides to parathyroid hormone affects its structure, potency, and transepithelial permeation. Bioconjug Chem 26:477–88. 10.1021/bc5005763 25611217

[CIT0050] Kwon M-K, Nam J-O, Park R-W, et al. (2008). Antitumor effect of a transducible fusogenic peptide releasing multiple proapoptotic peptides by caspase-3. Mol Cancer Ther 7:1514–22. 10.1158/1535-7163.MCT-07-2009 18566222

[CIT0051] Law B, Quinti L, Choi Y, et al. (2006). A mitochondrial targeted fusion peptide exhibits remarkable cytotoxicity. Mol Cancer Ther 5:1944–9. 10.1158/1535-7163.MCT-05-0509 16928814

[CIT0052] Lee J, Kim S, Shin DH, et al. (2011). Neuroprotective effect of Cu,Zn-superoxide dismutase fused to a TCTP-derived protein transduction domain. Eur J Pharmacol 666:87–92. 10.1016/j.ejphar.2011.05.040 21651901

[CIT0053] Levine S, Klaiber-Franco R, Paradiso PR. (1987). Demonstration that glycoprotein G is the attachment protein of respiratory syncytial virus. J Gen Virol 68: 2521–4. 10.1099/0022-1317-68-9-2521 3655746

[CIT0054] Li S, Ge F. (2017). Current understanding of the TCTP Interactome. Results Probl Cell Differ 64:127–36. 10.1007/978-3-319-67591-6_5[PMC][29149405] 29149405

[CIT0055] Liang JF, Yang VC. (2005). Insulin-cell penetrating peptide hybrids with improved intestinal absorption efficiency. Biochem Biophys Res Commun 335:734–8. 10.1016/j.bbrc.2005.07.142 16115469

[CIT0056] Lim S, Kim W, Kim Y, Choi J-M. (2012). Identification of a novel cell-penetrating peptide from human phosphatidate phosphatase LPIN3. Mol Cells 34:577–82. 10.1007/s10059-012-0284-y 23263658 PMC3887828

[CIT0057] Lönn P, Dowdy SF. (2015). Cationic PTD/CPP-mediated macromolecular delivery: charging into the cell. Expert Opin Drug Deliv 12:1627–36. 10.1517/17425247.2015.1046431 25994800

[CIT0058] MacDonald SM, Rafnar T, Langdon J, Lichtenstein LM. (1995). Molecular identification of an IgE-dependent histamine-releasing factor. Science 269:688–90. 10.1126/science.7542803 7542803

[CIT0059] Maeng J, Kim HY, Shin DH, Lee K. (2013). Transduction of translationally controlled tumor protein employing TCTP-derived protein transduction domain. Anal Biochem 435:47–53. 10.1016/j.ab.2012.11.029 23256924

[CIT0060] McCarthy S, Somayajulu M, Sikorska M, et al. (2004). Paraquat induces oxidative stress and neuronal cell death; neuroprotection by water-soluble Coenzyme Q10. Toxicol Appl Pharmacol 201:21–31. 10.1016/j.taap.2004.04.019 15519605

[CIT0061] McLellan JS, Ray WC, Peeples ME. (2013). Structure and function of respiratory syncytial virus surface glycoproteins. Curr Top Microbiol Immunol 372:83–104. 10.1007/978-3-642-38919-1_4[PMC][24362685] 24362685 PMC4211642

[CIT0062] Mondola P, Damiano S, Sasso A, Santillo M. (2016). The Cu, Zn superoxide dismutase: not only a dismutase enzyme. Front Physiol 7:594. 10.3389/fphys.2016.00594 PMC5126113 27965593

[CIT0063] Morishita M, Kamei N, Ehara J, et al. (2007). A novel approach using functional peptides for efficient intestinal absorption of insulin. J Control Release 118:177–84. 10.1016/j.jconrel.2006.12.022 17270307

[CIT0064] Muto K, Kamei N, Yoshida M, et al. (2016). Cell-penetrating peptide penetratin as a potential tool for developing effective nasal vaccination systems. J Pharm Sci 105:2014–7. 10.1016/j.xphs.2016.03.026 27155764

[CIT0065] Park J, Ryu J, Kim K-A, et al. (2002). Mutational analysis of a human immunodeficiency virus type 1 Tat protein transduction domain which is required for delivery of an exogenous protein into mammalian cells. J Gen Virol 83:1173–81. 10.1099/0022-1317-83-5-1173 11961273

[CIT0066] Patel LN, Zaro JL, Shen W-C. (2007). Cell penetrating peptides: intracellular pathways and pharmaceutical perspectives. Pharm Res 24:1977–92. 10.1007/s11095-007-9303-7 17443399

[CIT0067] Prochiantz A. (2000). Messenger proteins: homeoproteins, TAT and others. Curr Opin Cell Biol 12:400–6. 10.1016/s0955-0674(00)00108-3 10873818

[CIT0068] Prochiantz A, Joliot A. (2003). Can transcription factors function as cell-cell signalling molecules? Nat Rev Mol Cell Biol 4:814–9. 10.1038/nrm1227 14570063

[CIT0069] Richard JP, Melikov K, Brooks H, et al. (2005). Cellular uptake of unconjugated TAT peptide involves clathrin-dependent endocytosis and heparan sulfate receptors. J Biol Chem 280:15300–6. 10.1074/jbc.M401604200 15687490

[CIT0070] Rothbard JB, Jessop TC, Lewis RS, et al. (2004). Role of membrane potential and hydrogen bonding in the mechanism of translocation of guanidinium-rich peptides into cells. J Am Chem Soc 126:9506–7. 10.1021/ja0482536 15291531

[CIT0071] Ruseska I, Zimmer A. (2020). Internalization mechanisms of cell-penetrating peptides. Beilstein J Nanotechnol 11:101–23. 10.3762/bjnano.11.10 31976201 PMC6964662

[CIT0072] Schwarze SR, Ho A, Vocero-Akbani A, Dowdy SF. (1999). In vivo protein transduction: delivery of a biologically active protein into the mouse. Science 285:1569–72. 10.1126/science.285.5433.1569 10477521

[CIT0073] Skotland T, Iversen TG, Torgersen ML, Sandvig K. (2015). Cell-penetrating peptides: possibilities and challenges for drug delivery in vitro and in vivo. Molecules 20:13313–23. 10.3390/molecules200713313 26205056 PMC6332435

[CIT0074] Song Y, Wang Y, Thakur R, et al. (2004). Mucosal drug delivery: membranes, methodologies, and applications. Crit Rev Ther Drug Carrier Syst 21:195–256. 10.1615/critrevtherdrugcarriersyst.v21.i3.20 15248809

[CIT0075] Swanson JA. (2008). Shaping cups into phagosomes and macropinosomes. Nat Rev Mol Cell Biol 9:639–49. 10.1038/nrm2447 18612320 PMC2851551

[CIT0076] van den Berg A, Dowdy SF. (2011). Protein transduction domain delivery of therapeutic macromolecules. Curr Opin Biotechnol 22:888–93. 10.1016/j.copbio.2011.03.008 21489777

[CIT0077] Vargason AM, Anselmo AC, Mitragotri S. (2021). The evolution of commercial drug delivery technologies. Nat Biomed Eng 5:951–67. 10.1038/s41551-021-00698-w 33795852

[CIT0078] Vivès E, Brodin P, Lebleu B. (1997). A truncated HIV-1 Tat protein basic domain rapidly translocates through the plasma membrane and accumulates in the cell nucleus. J Biol Chem 272:16010–7. 10.1074/jbc.272.25.16010 9188504

[CIT0079] Wadia JS, Stan RV, Dowdy SF. (2004). Transducible TAT-HA fusogenic peptide enhances escape of TAT-fusion proteins after lipid raft macropinocytosis. Nat Med 10:310–5. 10.1038/nm996 14770178

[CIT0080] Wassarman PM. (2008). Zona pellucida glycoproteins. J Biol Chem 283:24285–9. 10.1074/jbc.R800027200 18539589 PMC2528931

[CIT0081] Wender PA, Mitchell DJ, Pattabiraman K, et al. (2000). The design, synthesis, and evaluation of molecules that enable or enhance cellular uptake: peptoid molecular transporters. Proc Natl Acad Sci U S A 97:13003–8. 10.1073/pnas.97.24.13003 11087855 PMC27168

[CIT0082] Yap MKK, Misuan N. (2019). Exendin-4 from Heloderma suspectum venom: from discovery to its latest application as type II diabetes combatant. Basic Clin Pharmacol Toxicol 124:513–27. 10.1111/bcpt.13169 30417596

[CIT0083] Yin Y, Li B, Zhou L, et al. (2020). Protein transduction domain-mediated influenza NP subunit vaccine generates a potent immune response and protection against influenza virus in mice. Emerg Microbes Infect 9:1933–42. 10.1080/22221751.2020.1812436 32811334 PMC8284974

[CIT0084] Yu J-R, Kim S, Lee J-B, Chang J. (2008). Single intranasal immunization with recombinant adenovirus-based vaccine induces protective immunity against respiratory syncytial virus infection. J Virol 82:2350–7. 10.1128/JVI.02372-07 18094185 PMC2258907

[CIT0085] Yusuf H, Kett V. (2017). Current prospects and future challenges for nasal vaccine delivery. Hum Vaccin Immunother 13:34–45. 10.1080/21645515.2016.1239668 27936348 PMC5287317

[CIT0086] Zhang X, Zhang X, Wang F. (2012). Intracellular transduction and potential of Tat PTD and its analogs: from basic drug delivery mechanism to application. Expert Opin Drug Deliv 9:457–72. 10.1517/17425247.2012.663351 22432469

[CIT0087] Ziegler A. (2008). Thermodynamic studies and binding mechanisms of cell-penetrating peptides with lipids and glycosaminoglycans. Adv Drug Deliv Rev 60:580–97. 10.1016/j.addr.2007.10.005 18045730

